# Biological Significance of Marine Actinobacteria of East Coast of Andhra Pradesh, India

**DOI:** 10.3389/fmicb.2017.01201

**Published:** 2017-07-06

**Authors:** Alapati Kavitha, Handanahal S. Savithri

**Affiliations:** Department of Biochemistry, Indian Institute of Science Bangalore, India

**Keywords:** marine actinobacteria, *Dietzia*, *Kocuria*, *Nocardiopsis*, *Streptomyces*

## Abstract

An attempt was made to identify actinobacterial strains present in the marine soil of East Coast regions *viz*., Chirala, Bapatla, and Peddaganjam, Andhra Pradesh; Kanyakumari, Tamil Nadu and Goa, Goa along with the study of their antimicrobial potential. Eight out of 73 actinobacterial strains isolated from these regions showed strong antimicrobial activity against Gram positive bacteria, Gram negative bacteria, and *Candida albicans*. Molecular identification (16S rRNA analysis) of the eight strains revealed that they belong to *Dietzia* sp., *Kocuria* sp., *Nocardiopsis* sp., and *Streptomyces* spp. ISP (International *Streptomyces* project) -1, ISP-2 and starch casein media supported high antimicrobial potential after 5–6 days of growth. Production of antimicrobials by the strains varied significantly with different carbon and nitrogen sources. Gas chromatography mass spectrometry (GCMS) analysis of volatile compounds produced by the strains illustrated an array of antimicrobial compounds such as 1, 2-benzene dicarboxylic acid, 2-piperidinone, pyrrolo[1,2-a]pyrazine-1,4-dion, phenyl ethyl alcohol, 3-phenyl propionic acid etc. Ours is the first report on the study and detection of above mentioned antimicrobial metabolites from *Dietzia* sp. (A3), *Kocuria* sp. (A5), and *Nocardiopsis* sp. (A7). By sequence based analysis for secondary metabolites, non-ribosomal peptide synthetase (NRPS) gene cluster was noticed in six strains (A2, A3, A4, A6, A7, and A8) and none of them had polyketide synthase (PKS) system. The present study intimates the biological potentiality of the actinobacterial strains isolated from East Coast of Andhra Pradesh, India which reveals further scope to investigate new bioactive compounds from them by employing both natural product chemistry and modern biotechnological aspects.

## Introduction

Pathogens causing infectious diseases are rapidly developing resistance towards traditional antibiotics (Chambers and DeLeo, 2009; Morens and Fauci, 2013; Ventola, 2015); therefore, there is an urgent necessity to search for safer and more potent compounds with broader spectrum of activity (Devine et al., 2017; WHO, 2017). Natural compounds or their derived products persist as a lead hub for the discovery of novel medicines to treat most of the human diseases. It has also been estimated that about 60% of the drugs that are available now including penicillin, anthracycline, bleomycins A_2_ and B_2_, mitomycin C, doxorubicin, epothilones, camptothecin, lovastatin etc. are mostly obtained from natural products (Lam, 2007; Newman, 2008; Cragg and Newman, 2013; Newman and Cragg, 2016). By knowing their potent bioactivities, organic chemists have developed new drugs using modern advancements in synthetic biology (Beghyn et al., 2008; Maier, 2015; Rodrigues et al., 2016).

Originally considered as an intermediate group between bacteria and fungi, Actinobacteria include Gram-positive bacteria with high G+C (>55%) content in their DNA which provide fifty percent of bioactive metabolites as recorded in the Dictionary of Natural Products (Barka et al., 2016). Initially, they were considered to be native to terrestrial habitats, but they are also common in marine ecosystems (Behie et al., 2017; Betancur et al., 2017) as evident by the isolation of various genera like *Agrococcus, Arthrobacter, Dietzia, Gordonia, Mycobacterium, Pseudonocardia, Rhodococci, Streptomyces* etc. (Claverias et al., 2015). The rate of finding new bioactive metabolites from the species of soil habitats has reduced. Therefore, a few attempts have been made to understand the microbial diversity of marine sediments which are an inexhaustible resource for the search of new drugs (Hassan et al., 2017).

Adaptation of marine actinobacteria to extreme climatic conditions such as high salinity, high pressure, and high temperature have modified their physiological conditions to survive and elaborate novel bioactive metabolites (Blunt et al., 2015, 2016, 2017; Behie et al., 2017; Kamjam et al., 2017). Approximately, 41 species belonging to 8 genera have been recorded from four states (Maharastra, Kerala, Tamil Nadu, and Andhra Pradesh) of Indian Peninsula (Sivakumar et al., 2007) and have been shown to yield new bioactive compounds (Ramesh and Mathivanan, 2009; Ramesh and William, 2012). East Coast regions of Andhra Pradesh, India have not been much explored for the presence of micro-organisms that could produce potent new drugs against several diseases. Therefore, an attempt was made to study the diversity of marine actinobacteria in East Coast as well as their antimicrobial potential.

## Materials and methods

### Collection of marine soil samples

Marine soil samples were collected at a depth of 14 cm from the surface of different marine habitats *viz*., Suryalanka (Bapatla region), Chirala, Peddapalem (Peddaganjam region) located near East Coast of Andhra Pradesh, India along with Kanyakumari, Tamil Nadu and Goa, Goa. They were air-dried and pretreated with calcium carbonate (1:1 w/w) followed by drying in a hot air oven at 45°C for 1 h, in order to reduce the contamination with bacteria and molds (El-Nakeeb and Lechevalier, 1963; Kavitha et al., 2010).

### Isolation of actinobacterial strains

International *Streptomyces* project (ISP-1, Tryptone glucose yeast extract) and ISP-2 (Yeast extract-malt extract-dextrose) agar media were prepared, sterilized at 121°C for 15 min and poured into Petri plates under aseptic conditions. Antibiotics such as streptomycin and amphotericin-B were added to the media just before pouring into Petri plates. Soil dilution plate technique was used for the isolation and enumeration of actinobacterial strains (Williams and Cross, 1971; Kavitha et al., 2010). Marine soil (1 g) sample pretreated with calcium carbonate was suspended in 100 ml of sterile distilled water followed by plating 0.1 ml of 10^−3^ serial dilution on different Petri dishes. After incubation of the plates at 30°C for 10 days, actinobacterial strains were isolated by observing the characteristics like tough and leathery colonies which are partially embedded into the agar (Jensen et al., 1991).

### Screening of actinobacterial strains for potent antimicrobial metabolites using agar well diffusion method

The secondary metabolites produced by the actinobacterial strains were extracted by using standard protocol (Ellaiah et al., 2005; Kavitha et al., 2010). Under aseptic conditions, actinobacterial strains were inoculated individually into the seed medium (ISP-2 broth) and incubated for 24 h. After that, the seed culture (10% v/v) was transferred into the production medium (ISP-2) and allowed to ferment for 5 days at 28 ± 2°C, 180 rpm. Sterile Whattman No. 1 filter papers were used to separate biomass from their culture filtrates. The collected biomass of all the strains was dried in a hot air oven and the residual dry weight was measured (mg/100 ml). The secondary metabolites obtained from all the culture filtrates were extracted with ethyl acetate twice, pooled individually and condensed under vacuum to yield solid residues. The residues were then resuspended in ethyl acetate and checked for their antimicrobial activity against the overnight grown cultures of *Bacillus megaterium* (NCIM 2187), *B. subtilis* (MTCC 441), *Staphylococcus aureus* (MTCC 96), *Pseudomonas aeruginosa* (MTCC 424) whereas 24–48 h old culture of *Candida albicans* (MTCC 183) was tested for evaluating antifungal activity.

The secondary metabolites produced by the strains were tested for their antimicrobial potentiality using agar well diffusion assay (Cappuccino and Sherman, 2002). Luria agar and Potato dextrose agar media were employed for the growth of test bacteria and fungus, respectively. About 0.1 ml of test bacterial/fungal suspension was transferred into the corresponding media (100 ml) sterilized previously at 15 lbs pressure (121°C) for 15 min. The inoculated medium was thoroughly mixed, poured into Petri plates and allowed to solidify under aseptic conditions. After that, wells of around 5 mm diameter were drilled into the agar medium with the help of a sterilized cork borer. The solvent extracts (50 μl) prepared at a concentration of 5 mg/ml were added to each well and ethyl acetate alone served as control. The inoculated plates were incubated at 30°C for 24 h and the diameter of inhibition zone was measured for bacteria and fungus.

Antimicrobial potential of the metabolites produced by the actinobacterial strains was examined to select the potent ones among the 73 isolated actinobacterial strains. Eight potent strains were chosen to determine their taxonomic position through cultural and molecular 16S rRNA gene fragment analysis.

### Taxonomic studies of the eight actinobacterial strains

Cultural and molecular (16S rRNA gene sequencing) analysis of the strains were studied. Different ISP media *viz*., ISP-1 (Tryptone-yeast extract agar), ISP-2 (Yeast extract malt extract dextrose agar), ISP-3 (Oat meal agar), ISP-4 (Inorganic salts starch agar), ISP-5 (Glycerol-asparagine salts agar), ISP-6 (Peptone yeast extract iron agar medium), and ISP-7 (Tyrosine agar) as well as non ISP media like Czapek Dox, Gauze, Maltose tryptone, Nutrient, and Potato dextrose agar with initial pH 7.2 maintained at 30°C were used to monitor the characteristics of the organisms (Dietz and Theyer, 1980). Cultural characters including growth, color of the aerial mycelia and substrate mycelia with their pigmentation were recorded.

### Molecular identification of the potent actinobacterial strains through genomic (16S rRNA gene fragment) analysis

For the extraction of genomic DNA (gDNA), the cell mass of the strains grown individually in maltose tryptone broth at 30°C for 2–3 days was centrifuged at 10,000 rpm, 4°C for 20 min (Stach et al., 2003). The cells were resuspended immediately in 5 ml of TE25S buffer followed by rapid vortexing. The cells were exposed to heat shock treatment (kept in water bath at 90°C for 5–10 min with immediate cooling on ice) followed by the addition of 250 μl of lysozyme (100 mg/ml) for proper cell wall lysis. Two microliters of RNase A (20 mg/ml) was also added to this mixture and incubated at 37°C for 1–2 h with proper inversion at every 20 min. To this, 100 μl of pronase (20 mg/ml) and 300 μl of 10% SDS were added, mixed by inversion occasionally and incubated at 55°C for 2–3 h. Further, 1 ml of 5M NaCl and 325 μl of 10% CTAB were added, mixed thoroughly and incubated for 10 min at 65°C. Later, chloroform: isoamyl alcohol (24:1) solvent system was added to the mixture and incubated for 30 min at 37°C. The aqueous phase obtained after centrifugation at 8,000 rpm for 5 min was transferred to a fresh tube. An equal volume of ice-cold isopropanol was added to the aqueous phase, mixed by gentle inversion for 10 min at 37°C and centrifuged at 12,000 rpm, 4°C for 10 min. The white DNA precipitate obtained was washed with 1 ml of ice-cold 70% ethanol followed by another centrifugation step to remove excess ethanol. The pellet was air-dried and 100 μl of sterile H_2_O or 0.2X TE buffer was added to dissolve the DNA. The same procedure was followed for all the strains to get good quality of gDNA for further amplification of 16S rRNA region.

The 16S rRNA gene segment of actinobacterial strains was amplified by polymerase chain reaction (PCR) individually in a reaction mixture containing 1X PCR buffer (ThermoFisher Scientific, USA), 2 U of *Taq* polymerase, each deoxynucleoside triphosphate at a concentration of 200 μM, 50–100 ng of gDNA, 20 μM of primer forward (5′- GAGTTTGATCCTGGCTCA -3′) and 20 μM of primer reverse (5′- ACGGCTACCTTGTTACGACTT -3′). The final volume of the PCR mixture was made up to 100 μl by adding distilled H_2_O and the reaction mixture was overlaid with 80 μl of sterile mineral oil. Thermal cycling was carried out with a model S1000 (Bio-Rad, USA) and all the samples were subjected to an initial denaturation (3 min at 98°C) followed by denaturation (1 min at 94°C), annealing (1 min at 52°C, 28 consecutive cycles), extension (2 min at 72°C), and a final extension (5 min at 72°C) step at the end.

The amplified DNA fragment from all the strains was monitored on 1% agarose gel, eluted, and purified using Nucleospin gel extraction kit (Macherey-Nagel, Germany). The purified PCR products were sequenced using the Big-Dye terminator kit ABI 310 Genetic Analyzer (Applied Biosystems, USA) and further recorded their accession numbers by depositing them in National Center for Biotechnology Information (NCBI) GenBank. The 16S rRNA sequences were compared with that of related sequences obtained from GenBank through NCBI BLAST search program. Nucleotide substitution rates (Knuc values) were evaluated (Kimura, 1980). Phylogenetic tree using neighbor-joining method was constructed (Saitou and Nei, 1987) along with the statistical analysis of bootstrap values by employing Molecular Evolutionary Genetics Analysis (MEGA7) software (Thompson et al., 1997).

### Nutritional parameters affecting the production of antimicrobial metabolites

#### Growth pattern and effect of incubation time on the production of antimicrobial metabolites

Growth pattern of the eight actinobacterial strains and their antimicrobial activity against Gram positive bacteria (*B. megaterium* and *S. aureus*) and Gram negative bacteria (*Enterococcus faecalis* and *P. aeruginosa*) was recorded in ISP-2 medium for 8 days. Biomass was measured as dry weight of the cell mass (mg/100 ml culture medium) and the supernatant was extracted with ethyl acetate, vacuum dried in a rotavapor followed by testing the residues (1 mg/ml) for antimicrobial activity against bacteria through agar well diffusion method using the diameter of inhibition zone (mm) (Cappuccino and Sherman, 2002).

#### Effect of culture media composition on the production of antimicrobial metabolites

Effect of growth media on the production of antimicrobial metabolites was studied by culturing the strains separately in different media *viz*., Arginine-glycerol (Arg-Gly), Czapek-Dox (Dox), ISP-1, ISP-2, ISP-4, Luria broth (LB), Maltose-tryptone (MT), Nutrient broth (NB), Starch-casein (SC), Yeast mannitol broth (YMB), and Yeast extract-peptic digest of animal tissue-dextrose (YPD) broths. Efficiency of the secondary metabolites of the strains was recorded as antimicrobial potential against Gram positive and Gram negative bacteria by employing agar well diffusion assay. The medium in which the strain elaborates maximum levels of antimicrobials was studied individually for all the eight strains (Kavitha and Vijayalakshmi, 2009).

#### Influence of carbon and nitrogen sources on the yield of antimicrobial metabolites

Various carbon sources such as dextrose, galactose, glycerol, maltose, mannitol, starch, sucrose, and xylose were added to the optimized media by replacing their carbon source. Likewise, the impact of different nitrogen sources on the yield of antimicrobials of the strains was studied by supplementing the nitrogen source in the medium with different nitrogen sources like ammonium chloride, aspartic acid, L-arginine, potassium nitrate, tryptone, urea, and yeast extract in the optimized carbon medium (Kavitha and Vijayalakshmi, 2009).

### Detection of possible secondary metabolites through gas chromatography—mass spectral analysis (GC-MS) and screening of biosynthetic gene clusters

#### Analysis of volatile compounds by GCMS

The components of the biologically active crude extracts obtained from the strains were analyzed through Agilent GC-MS apparatus (GC: 7890A; MSD5975C). It has fused- silica HP-5 capillary column (30 m–0.25 mm, ID, film thickness of 0.25 mm) coupled directly with single quadrupole MS. Flow rate of the carrier gas, helium was maintained at 1 ml/min. Oven temperature was automated (50°C for 1 min, then 50–300°C at a rate of 5°C/min) and subsequently, held isothermally for 5 min. The temperature of injector port was kept at 250°C and that of transfer line at 300°C. MS quadrupole and MS source temperatures were maintained at 150 and 230°C, respectively (Boussaada et al., 2008). The peaks detected in GC were concluded as corresponding compounds through mass spectral data analysis software and NISTMS library data, 2008.

#### Preliminary detection of non-ribosomal peptide synthetases (NRPS) and type I polyketide synthases (PKS-I) biosynthetic gene systems

NRPS and PKS-I are the major biosynthetic gene sequences in micro-organisms involved for the production of bioactive polyketide and peptide compounds. Therefore, gDNA of the eight strains was checked for the presence or absence of these biosynthetic systems using actinobacterial specific NRPS and PKS-I PCR primers (Ayuso-Sacido and Genilloud, 2005). A 50 μl reaction cocktail containing 5 μl gDNA as template, 0.4 μM of each primer [A3F (5′ GCSTACSYSATSTACACSTCSGG 3′) and A7R (5′ SASGTCVCCSGTSCGGTAS 3′), for NRPS; K1 (5′ TSAAGTCSAACATCGGBCA 3′), and M6R (5′ CGCAGGTTSCSGTACCAGTA 3′) for PKS-I], 0.2 mM of each of the four dNTPs, 1 U *Taq* polymerase, 10X *Taq* buffer, and 10% DMSO was prepared for all the strains separately. PCR amplification protocol for the excepted product size included initial denaturation (95°C for 5 min) followed by 35 cycles of denaturation (95°C for 30 sec), annealing (59°C for 2 min—NRPS and 58°C for 2 min—PKS-I), and extension (72°C for 4 min). The amplified PCR products obtained after a final extension step at 72°C for 10 min were monitored on 1% (w/v) agarose gel electrophoresis.

## Results and discussion

### Enumeration of actinobacterial strains isolated from different marine soil samples and testing the efficiency of their antimicrobials

**Figure 1:**
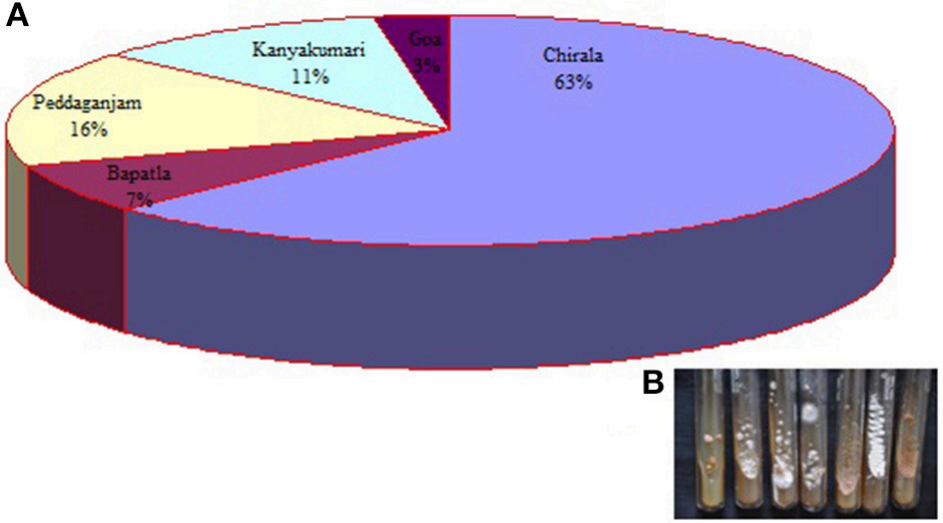
**(A)** Pie diagram illustrating the percentile of actinobacterial strains isolated from marine soil samples of Chirala, Bapatla, Peddaganjam, Andhra Pradesh; Kanyakumari, Tamil Nadu and Goa, Goa, **(B)** Image of few actinobacterial strains.

**Figure 2:**
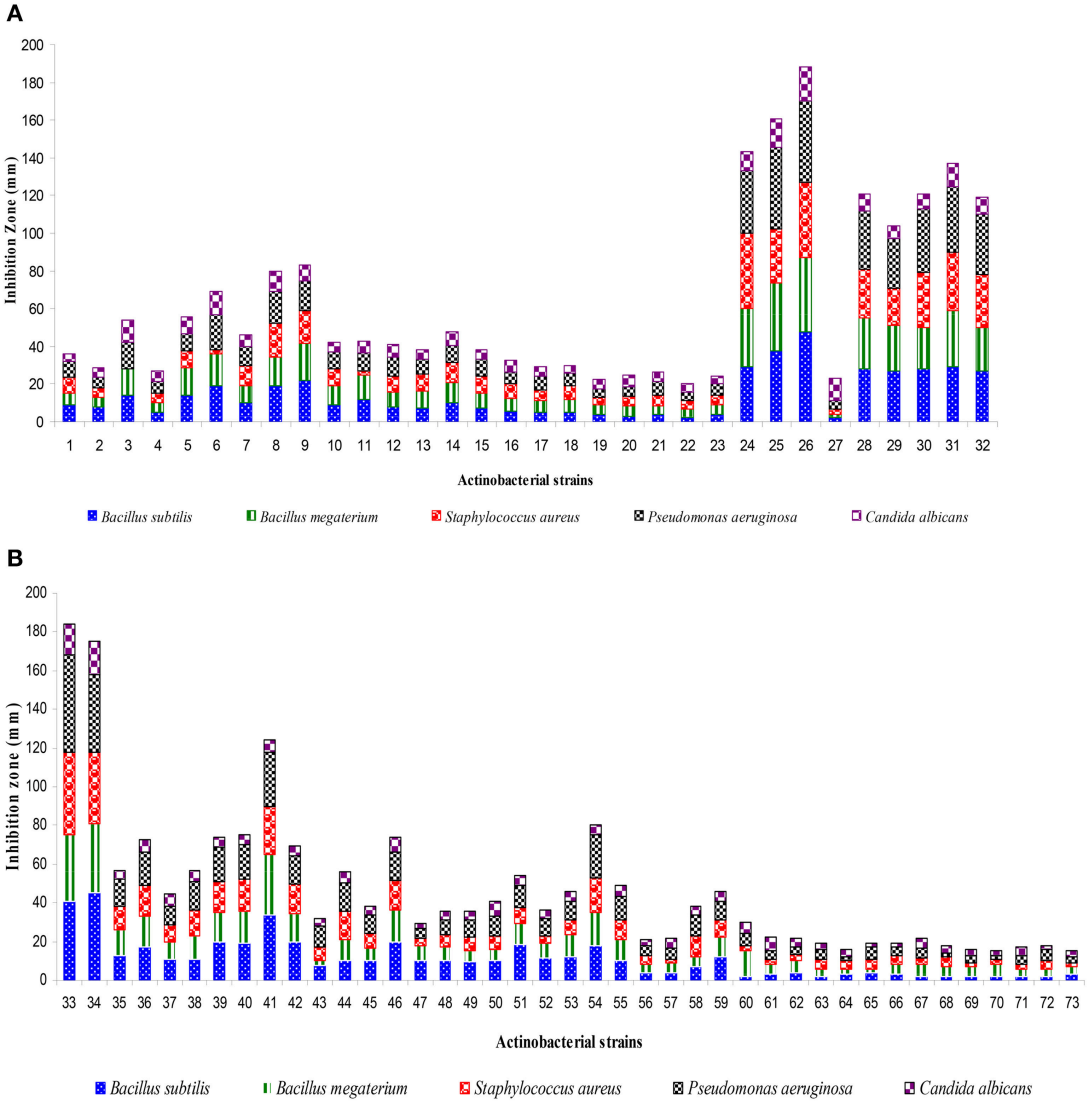
Screening of actinobacterial strains (1–73) for antimicrobial metabolites by using agar well diffusion assay. **(A)** Strains 1–32, **(B)** Strains 33–73. Basing on antimicrobial profile, actinobacterial strains *viz*., 24, 25, 26, 28, 30, 31, 33, and 34 were selected for further study and designated as A1–A8. Data was statistically analyzed by One-way ANOVA and found to be significant at 5% level (*P* < 0.05) between the strains.

A total of 73 actinobacterial strains were isolated from soil samples of East Coast of Indian marine ecosystem *viz*., Chirala, Bapatla, Peddaganjam, Andhra Pradesh along with Kanyakumari, Tamil Nadu and Goa, Goa. As depicted in Figure 1, majority of the actinobacterial strains were obtained from the marine soil samples of Chirala (63%) followed by Peddaganjam (16%) by serial dilution method on ISP-2 medium supplemented with 3% (w/v) NaCl. All the 73 actinobacterial strains isolated from the different marine soil samples were evaluated for their antimicrobial activity as described in Materials and Methods Section (Figures 2A,B). Among those, strains 24, 25, 26, 28, 30 (strains from Chirala region), 31 (strain from Bapatla region), 33 and 34 (strains from Peddaganjam region) exhibited high antimicrobial activity against the bacteria tested.

Marine ecosystem serves as an attractive source for the isolation and production of bioactive compounds (Blunt et al., 2015, 2016, 2017; Kamjam et al., 2017), bioactive pigments (Soliev et al., 2011), enzymes (Ramesh and William, 2012; Leipoldt et al., 2015), biofuels (Lewin et al., 2016), and also showed potential for biomineralization activities along with the maintenance of nutrient web cycle, biological N_2_ fixation, and environmental protection (Das et al., 2006; Biswas and Gresshoff, 2014; Alvarez et al., 2017). Around 9% actinobacteria was recorded in marine sediments (Bull et al., 2005) and suggested as a stable and prominent bioactive group of micro-organisms in marine ecology from the earlier findings (Claverias et al., 2015; Betancur et al., 2017). The present study also highlights the existence of numerous actinobacterial strains with various bioactivities from different marine coastal regions of India. Eight out of 73 strains (24, 25, 26, 28, 30, 31, 33, and 34) isolated from East Coast of Andhra Pradesh had shown pronounced bioactivity and designated them as A1, A2, A3, A4, A5, A6, A7, and A8 for further taxonomic and nutritional studies.

### Cultural and molecular identification of actinobacterial strains

Growth and cultural characteristics of the eight actinobacterial strains were studied on ISP and non-ISP agar media (Table 1). All the strains exhibited good to moderate to poor growth patterns on different media tested. Strains A1, A3, A4, A5, and A6 showed white to pink aerial mycelia with brown substrate mycelia whereas the strains A2 and A7 exhibited creamy aerial mycelia and dark brownish substrate mycelia. Diffused yellow pigmentation was observed with only strain A8, having white to light yellowish aerial mycelia and brown substrate mycelia. Actinobacteria are ubiquitous in nature and includes a wide range of color series ranging from gray, white, yellow, red, green, blue to black (Barka et al., 2016).

**Table 1:** Cultural characteristics of the actinobacterial strains (A1-A8) on different culture media.

**Actinobacterial strains**		**Cultural characters on different media**												
		**1**	**2**	**3**	**4**	**5**	**6**	**7**	**8**	**9**	**10**	**11**	**12**	**13**
A1	G	Good	Good	Good	Good	Good	Good	Good	Good	Good	Good	Good	Good	Good
	A	Cream	Cream	Dark cream	White	White	White	Dark cream	White	Cream	White	Light pink	Cream	Light pink
	R	Light brown	Cream	Light brown	Light brown	Light brown	White	Brown	Light brown	Cream	Light brown	Creamy brown	Light brown	Light brown
	P	–	–	–	–	–	–	–	–	–	–	–	–	–
A2	G	Good	Good	Good	Good	Good	Poor	Good	Good	Good	Moderate	Good	Good	Good
	A	Cream	Cream	Cream	Cream	Cream	Cream	White	Cream	Cream	Cream	White	Cream	Cream
	R	Creamy brown	Creamy brown	Reddish brown	Dark brown	White	White	Brown	White	Light brown	White	Dark brown	Creamy brown	Black
	P	–	–	–	–	–	–	–	–	–	–	–	–	–
A3	G	Good	Good	Good	Moderate	Good	Good	Good	Good	Good	Good	Good	Good	Good
	A	Light pink	Light pink	Light pink	White	Cream	White	Creamy brown	Dark brown	Cream	White	Light pink	Light brown	Pink
	R	Light brown	Light brown	Creamy brown	White	Cream	White	Brown	Black	Brown	Light brown	Light brown	Brown	Brown
	P	–	–	–	–	–	–	–	–	–	–	–	–	–
A4	G	Good	Good	Good	Good	Good	Moderate	Good	Good	Good	Moderate	Good	Good	Good
	A	Light pink	Light pink	Light pink	Cream	Cream	White	Dark pink	Dark pink	Dark pink	Light pink	Light pink	Light pink	Dark pink
	R	Brown	Brown	Dark brown	Cream	Light brown	White	Reddish brown	Reddish brown	Reddish brown	Dark brown	Dark brown	Creamy brown	Dark brown
	P	–		–	–	–	–		–	–	–	–	–	–
A5	G	Good	Good	Good	Good	Moderate	Good	Good	Good	Good	Good	Good	Good	Good
	A	Cream	Cream	Light yellow	White	White	White	White	White	White	White	Light pink	White	Light yellow
	R	Light brown	Cream	Creamy brown	White	White	White	Light brown	White	White	White	Light brown	Light brown	Yellow
	P	–	–	–	–	–	–	–	–	–	–			–
A6	G	Good	Good	Good	Poor	No	No	Good	No	Good	No	Good	Poor	Good
	A	Light pink	Cream	Light pink	Light pink	–	–	Dark cream	–	Light brown	–	Light pink	Light pink	Pink
	R	Light brown	Light brown	Dark brown	Dark brown	–	–	Dark brown	–	Brown	–	Dark brown	Dark brown	Dark brown
	P	–	–	–	–	–	–	–	–	–	–	–	–	–
A7	G	Good	Moderate	Good	Good	Good	Good	Good	Good	Good	Good	Good	Good	Good
	A	Cream	Cream	Cream	Cream	Cream	Cream	Cream	Cream	Cream	Cream	Cream	Cream	Cream
	R	Dark brown	Light brown	Dark brown	Dark brown	Dark brown	Dark brown	Dark brown	Dark brown	Dark brown	Dark brown	Dark brown	Dark brown	Dark brown
	P	–	–	–	–	–	–	–	–	–	–	–	–	–
A8	G	Good	Moderate	Good	Good	Good	Good	Good	Moderate	Moderate	Good	Good	Good	Good
	A	White	White	White	White	White	White	White	White	White	White	White	White	White
	R	Yellow to light brown	Light brown	Yellow to light brown	Yellow to light brown	Yellow to light brown	Yellow to light brown	Yellow to light brown	Light brown	Light brown	Yellow to light brown	Yellow to light brown	Yellow to light brown	Yellow to light brown
	P	Yellow	Yellow	Yellow	Yellow	Yellow	Yellow	Yellow	Yellow	Yellow	Yellow	Yellow	Yellow	Yellow

*G, Growth; A, Aerial mycelium; R, Reverse mycelium; P, Pigment production. Media—1 International Streptomyces Project (ISP) agar 1, 2 ISP-2 agar, 3 ISP-3 agar, 4 ISP-4 agar, 5 ISP-5 agar, 6 ISP-6 agar, 7 ISP-7 agar, 8 Asparagine glucose agar, 9 Czapek-Dox agar, 10 Gauze agar, 11 Maltose tryptone agar, 12 Nutrient agar, 13 Potato dextrose agar*.

**Figure 3:**
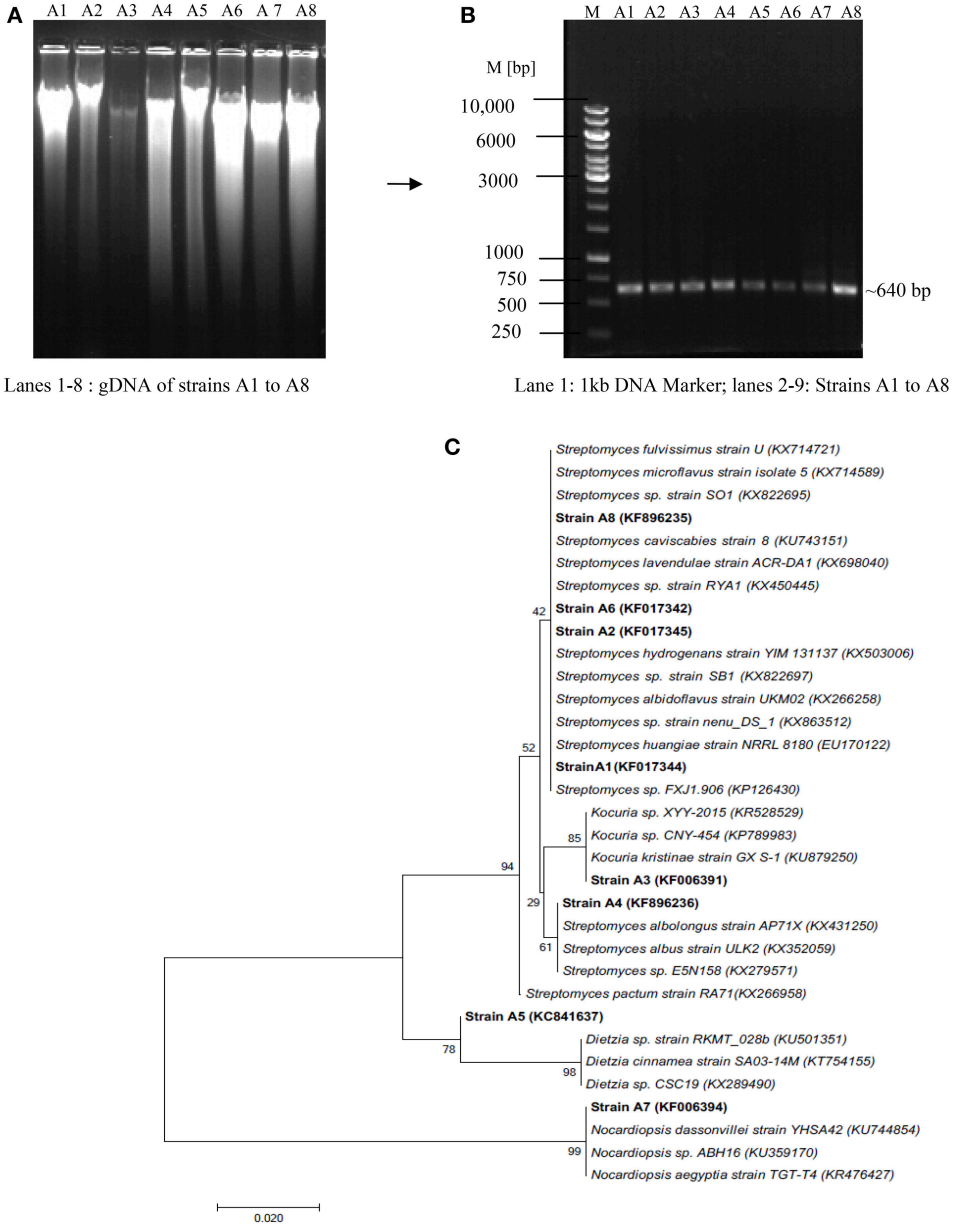
Molecular identification of actinobacterial strains through 16S rRNA gene fragment analysis **(A)** Isolation of Genomic DNA, **(B)** PCR amplification of gDNA with Act 254F and 894R primers **(C)** Phylogenetic tree showing taxonomic position of the isolated actinoabcterial strains with respect to the sequences deposited in NCBI Genbank.

Molecular identification of the strains was carried out through 16S rRNA gene fragment analysis, a powerful tool to recognize micro-organisms up to genus level (Barka et al., 2016). Figures 3A,B depicts the isolation of gDNA from all the strains and amplification with actinobacterial specific 16S rRNA gene fragment primers as described in Section Materials and Methods. The amplified products were sequenced and the phylogenetic position of the strains was analyzed through NCBI Blast search program. The results revealed that the strains *viz*., A3, A5, and A7 belong to genera *Dietzia, Kocuria*, and *Nocardiopsis* with 98, 99, and 100% similarity, respectively; while the remaining ones A1, A2, A4, A6, and A8 showed similarity index between 97 and 100% to the genus *Streptomyces* (Figure 3C). The 16S rRNA gene sequence of all the strains has been deposited in NCBI Genbank with the accession numbers KF017344 (A1—*Streptomyces* sp.), KF017345 (A2—*Streptomyces* sp.), KF006391 (A3—*Kocuria* sp.), KF896236 (A4—*Streptomyces* sp.), KC841637 (A5—*Dietzia* sp.), KF017342 (A6—*Streptomyces* sp.), KF006394 (A7—*Nocardiopsis* sp.), and KF896235 (A8—*Streptomyces* sp.).

### Nutritional parameters affecting the production of antimicrobial metabolites

**Figure 4:**
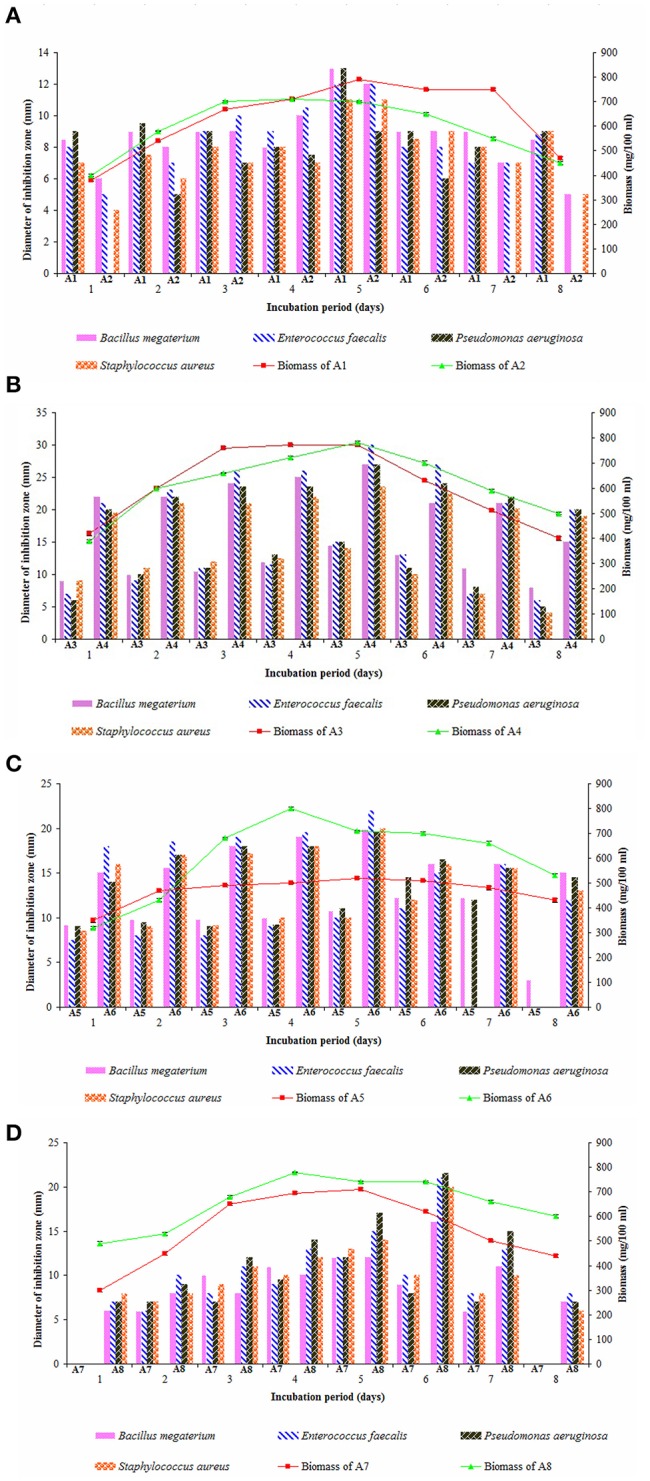
Growth pattern of actinobacterial strains and their antimicrobial potential in ISP-2 medium **(A)** A1 and A2, **(B)** A3 and A4, **(C)** A5 and A6, and **(D)** A7 and A8. Actinobacterial strains exhibited iodiophase between 4th and 5th (A2, A3, A4, and A7), 5th and 6th (A5, A6, and A8), and 5th–7th days (A1) of growth period. The secondary metabolites obtained from 5-day old cultures of A1, A2, A3, A4, A6, and A7 showed high antimicrobial activity against Gram-positive bacteria and Gram-negative bacteria while those obtained from 6 days of the strains A5 and A8 had pronounced effect. Data was statistically analyzed by Two-way ANOVA and had no significant difference between the strains.

Secondary metabolites especially antimicrobial compounds produced during iodiophase are the natural missiles to combat infectious diseases. Elaboration of secondary metabolites is highly dependent on the growth and nutritional parameters (Gonzalez et al., 2003; Jose et al., 2013). Growth pattern of the eight strains studied individually in ISP-2 medium for 8 days and their interesting antimicrobial profiles were tested against Gram positive and Gram negative bacteria by employing agar well diffusion assay (Figures 4A–D). Log phase of all the strains appeared within 48 h of growth period. Strains A2, A3, A4, and A7 entered stationary phase on 4th and 5th days of incubation and then declined while the strains A5, A6, and A8 exhibited stationary growth on 5th and 6th days. Strain A1 showed a little bit prolonged stationary phase between 5th and 7th days of growth.

In most of the actinobacterial strains, production of antimicrobials happens mostly after 4–6 days of growth (Kavitha and Vijayalakshmi, 2009; Kavitha et al., 2010) and those recorded from 5 days old culture of *Streptomyces* spp. showed best potential (Manivasagan et al., 2014). In the present study, the ethyl acetate extracts obtained from 5th day old cultures of the strains A1, A2, A3, A4, A6, and A7 exerted high antimicrobial activity against the organisms tested whereas the strains A5 and A8 produced better yields of antimicrobials after 6 days of incubation. Out of the eight strains, secondary metabolites obtained from the strain A4 (*Streptomyces* sp.) were highly active on *E. faecalis* followed by those of strain A6 (*Streptomyces* sp.). Ethyl acetate extracts of strains A8 (*Streptomyces* sp.) and A5 (*Dietzia* sp.) are more effective against *P. aeruginosa*. Other strains, A3 (*Kocuria* sp.) and A7 (*Nocardiopsis* sp.) showed more or less similar antimicrobial pattern on all the bacteria tested. Claverias et al. (2015) recorded the antimicrobial profile of marine actinobacteria including *Streptomyces* and *Dietzia* isolated from Valparaiso bay, Chile. Kamjam et al. (2017) reviewed the bioactive compounds produced by deep sea *Streptomyces* spp. and *Nocardiopsis* spp. To our knowledge, this is the first report on the antimicrobial profile of a marine strain A3 (*Kocuria* sp.).

**Figure 5:**
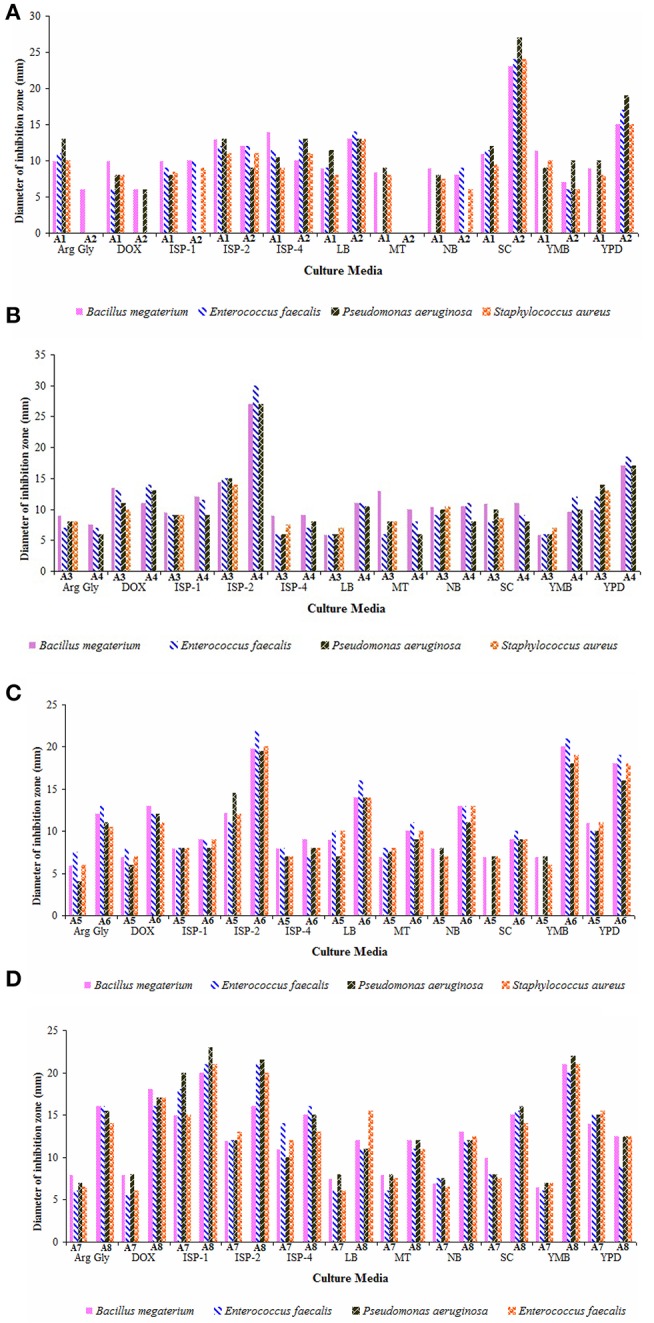
Effect of culture media on the yields of antimicrobial metabolites of actinobacterial strains **(A)** A1 and A2, **(B)** A3 and A4, **(C)** A5 and A6, and **(D)** A7 and A8. Arginine-glycerol (Arg-Gly), Czapek-Dox (Dox), ISP-1, ISP-2, ISP-4, Luria broth (LB), Maltose-tryptone (MT), nutrient broth (NB), Starch-casein (SC), Yeast mannitol broth (YMB), and Yeast extract-peptic digest of animal tissue-dextrose (YPD) broths. ISP-2 medium supported better yields of antimicrobial metabolites for majority of the strains (A1, A3, A4, A5, and A6) whereas the strain A2 preferred starch casein broth for its antimicrobial activity. Other strains, A7 and A8 elaborated high antimicrobial activity when grown in ISP-1 medium. Data was statistically analyzed by Two-way ANOVA and found to be significant at 5% level (*P* < 0.05) between the strains.

Nutritional parameters greatly influence the biosynthesis of antimicrobial compounds which may be varied for different strains. Therefore, the secondary metabolites produced by eight strains in 11 different growth media are illustrated (Figures 5A–D). ISP-2 medium served as the best culture medium for the production of antimicrobial metabolites by the strains A1, A3, A4, A5, and A6. High yields of antimicrobials were observed for the strains A7 and A8 when cultured in ISP-1 medium. Starch-casein broth supported high antimicrobial activity against the test bacteria for strain A2 followed by YPD. Other media like Czapek-Dox (for strain A3), YPD (for strains A5 and A7), ISP-5 and Starch-casein (for strain A1), YMB and YPD (for strain A6), YMB and ISP-2 (for strain A8) stood next preferential ones for the production of antimicrobial metabolites. Among all, strains A2 and A4 showed potent antimicrobial activity against the bacteria tested. Earlier findings also illustrated ISP-2 (Rateb et al., 2014) and starch casein as suitable media for the production of secondary metabolites by *Streptomyces* spp. (Djinni et al., 2013).

**Figure 6:**
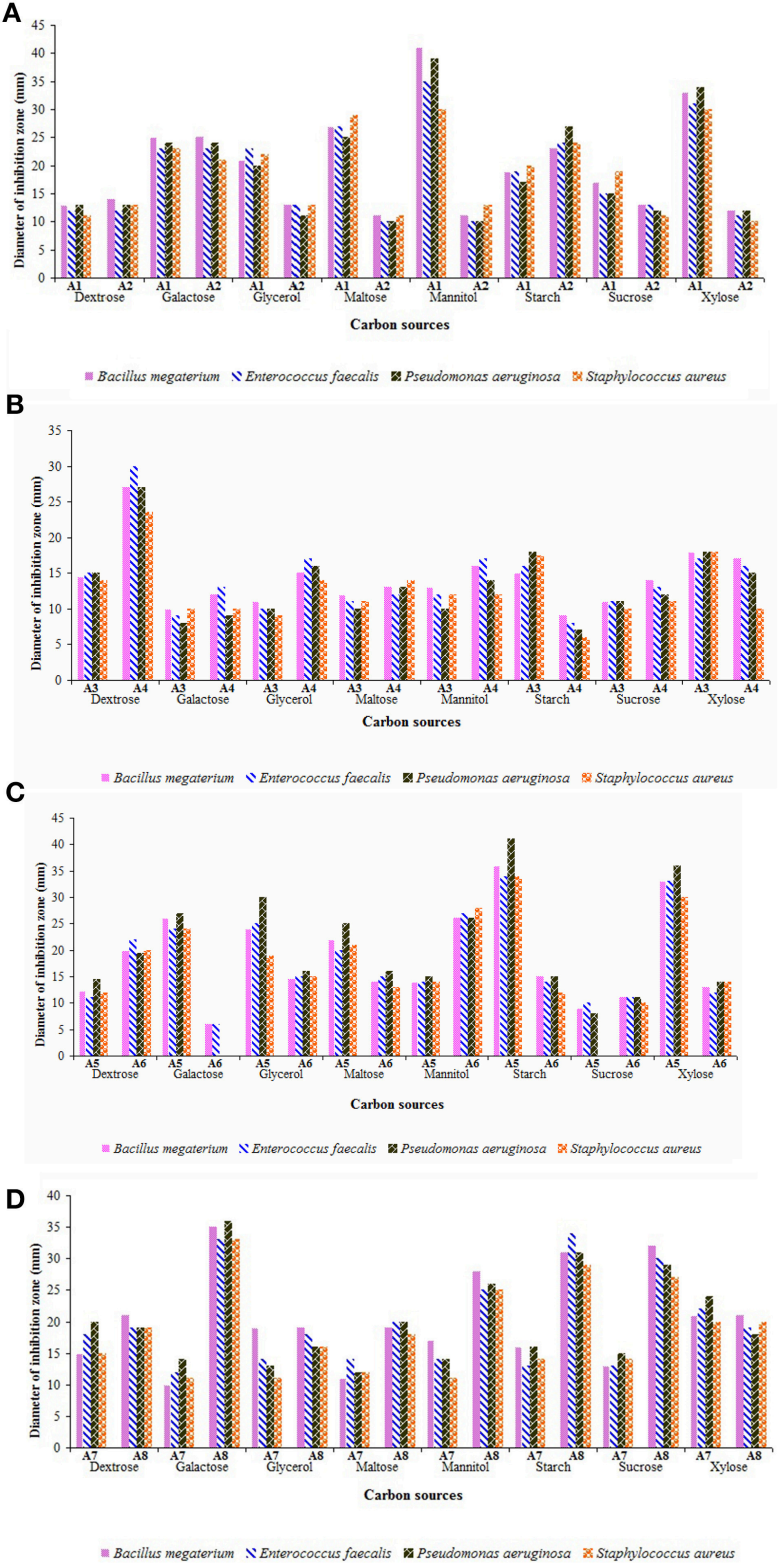
Influence of carbon sources on the yields of antimicrobial metabolites of actinobacterial strains **(A)** A1 and A2, **(B)** A3 and A4, **(C)** A5 and A6, and **(D)** A7 and A8. Utilization of carbon sources by the strains varied widely. Optimal culture medium with preferred carbon sources such as mannitol (for strains A1 and A6), starch and galactose (for strain A2), xylose (for strains A3 and A7), dextrose (for strain A4), starch (for strain A5), and galactose (for strain A8) favored strong antimicrobial potential. Data was statistically analyzed by Two-way ANOVA and found to be significant at 5% level (*P* < 0.05) between the strains.

Synthesis of antibiotics depends on the type of nutrients amended in the culture media. Especially, carbon and nitrogen sources play a crucial role on the biosynthesis of secondary metabolites by the strains both at level of activity and over expression of the genes corresponding to the enzymes involved (Sanchez et al., 2010). Initially, production of antimicrobial metabolites by the strains in the selected media supplemented with different carbon sources were tested (Figures 6A–D). The secondary metabolites of the strain A1 showed promising antimicrobial activity against the bacteria tested when cultured in ISP-2 medium amended with mannitol followed by xylose, maltose, and galactose. Starch and galactose acted as promising carbon sources for the production of antimicrobial metabolites by the strain A2. Strains A3 and A7 showed optimal rates of secondary metabolites when grown in xylose enriched ISP-2 and ISP-1 media, respectively. Among the carbon sources tested, ISP-2 medium with dextrose remained as the best carbon source for the strain A4 to yield optimal antimicrobial metabolites. Strain A5 produced optimal yields of antimicrobial metabolites in the medium with starch followed by xylose. High levels of secondary metabolites were produced by the strain A6 in ISP-2 medium incorporated with mannitol whereas dextrose in ISP-1 medium replaced with other carbon sources like galactose followed by starch and sucrose enhanced the synthesis of antimicrobials of the strain A8. Out of all the strains, strains A5 (*Dietzia* sp.), A8 and A1 (*Streptomyces* spp.) exhibited strong antimicrobial potential. Among the bacteria tested, *P. aeruginosa* showed maximum sensitivity to the secondary metabolites of the strains A5 followed by A8 and A1.

The yield of secondary metabolites varied with different carbon sources in different strains. Dextrose enriched culture medium supported the production of secondary metabolites by strain A4 in agreement with the earlier reports on marine *Streptomyces* sp. (Manikkam et al., 2015; Haque et al., 2017). But, indeed it was less efficient in increasing the yields of secondary metabolites by most of the strains, when compared to that of other sugars. It can be inferred from these results that the simple sugars like dextrose are easily metabolized and utilized rapidly in the early log phase itself, whereas the other sugars may favor the growth of the organisms even up to stationary phase for the optimal release of secondary metabolites. Sengupta et al. (2015) also recorded that most of the actinobacterial strains isolated from Sundarbans mangrove ecosystem preferred polysaccharides (D-galactose and D-raffinose) over monosaccharides for antibiotic production. Sunita et al. (2015) observed maximum yields of antimicrobial metabolites by *Streptomyces* spp. in starch enriched medium.

**Figure 7:**
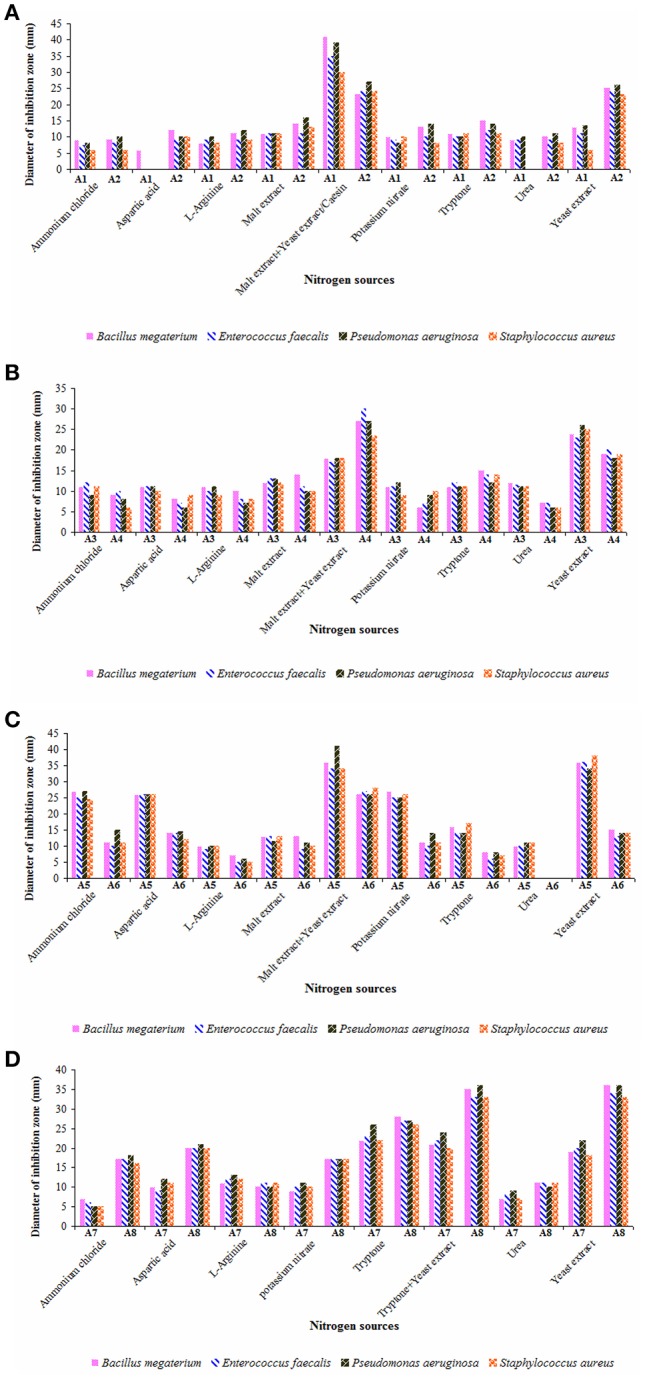
Impact of nitrogen sources on the yields of antimicrobial metabolites of actinobacterial strains **(A)** A1 and A2, **(B)** A3 and A4, **(C)** A5 and A6, and **(D)** A7 and A8. Actinobacterial strains exhibited strong antimicrobial potential when grown individually in the selected culture medium with malt extract plus yeast extract (strains A1, A4, and A6), casein/yeast extract (strain A2), yeast extract (strain A3), malt extract plus yeast extract/yeast extract alone (strain A5), and tryptone alone/tryptone plus yeast extract/yeast extract alone (strains A7 and A8). Data was statistically analyzed by Two-way ANOVA and found to be significant at 5% level (*P* < 0.05) between the strains.

Utilization of different nitrogen sources in the growth medium by actinobacterial strains are critical for secondary metabolite production (Francois and Stephane, 2001; Yao and Ye, 2016). Hence, the effect of various nitrogen sources on the yields of antimicrobial metabolites of the strains was presented in Figures 7A–D. Out of nine nitrogen sources, malt extract and yeast extract served as suitable combination in ISP-2 medium for the elaboration of antimicrobial metabolites by the strains A1, A4, and A6. Strain A2 exhibited high antimicrobial activity when cultured in starch casein medium as well as with yeast extract amendement. Strain A3 yielded optimal levels of antimicrobials in ISP-2 medium incorporated with only yeast extract (minus malt extract combination) whereas malt extract plus yeast extract and yeast extract alone favored high rates of secondary metabolites from the strain A5. Other nitrogen sources like potassium nitrate, ammonium chloride and aspartic acid also supported better yields of metabolites for the latter ones. ISP-1 media containing tryptone alone or tryptone plus yeast extract or yeast extract alone were quite suitable for the production of antimicrobial metabolites by the strains A7 and A8.

Rateb et al. (2014) isolated trirandamycin, an antifilarial drug lead from *Streptomyces* sp. 17,944 cultured in traditional ISP-2 (containing both malt extract and yeast extract) as well as under optimized conditions. Other researchers (Kavitha and Vijayalakshmi, 2009; Ripa et al., 2009) also proved **y**east extract as the best nitrogen source for antimicrobial metabolites. While screening *Streptomyces* spp. for the elaboration of antimicrobial metabolites. Khaliq et al. (2013) reported tryptone enriched medium for one of the strains SK-5. In the present study, the antimicrobial metabolites of the strains A5 and A8 were highly effective against *P. aeruginosa* whereas those produced by the strain A1 are more active on *B. megaterium*. Ours is the first report on the production and optimization of antimicrobial metabolites by *Dietzia* sp. (A3) and *Kocuria* sp. (A5). Secondary metabolites of other strains (A1, A2, A4, A6, and A8) belonging to *Streptomyces* spp. and *Nocardiopsis* sp. (A7) showed strong antimicrobial potential as evident from the earlier findings (Manivasagan et al., 2014; Kamjam et al., 2017).

### Analysis of volatile compounds from biologically active crude ethyl acetate extracts of the strains by GCMS

**Figure 8:**
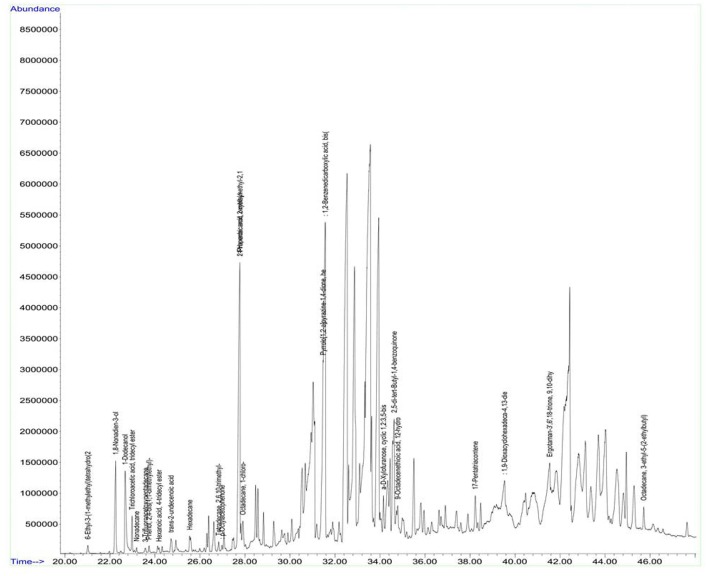
GC chromatogram of the secondary metabolites of *Nocardiopsis* sp. (A7).

**Figure 9:**
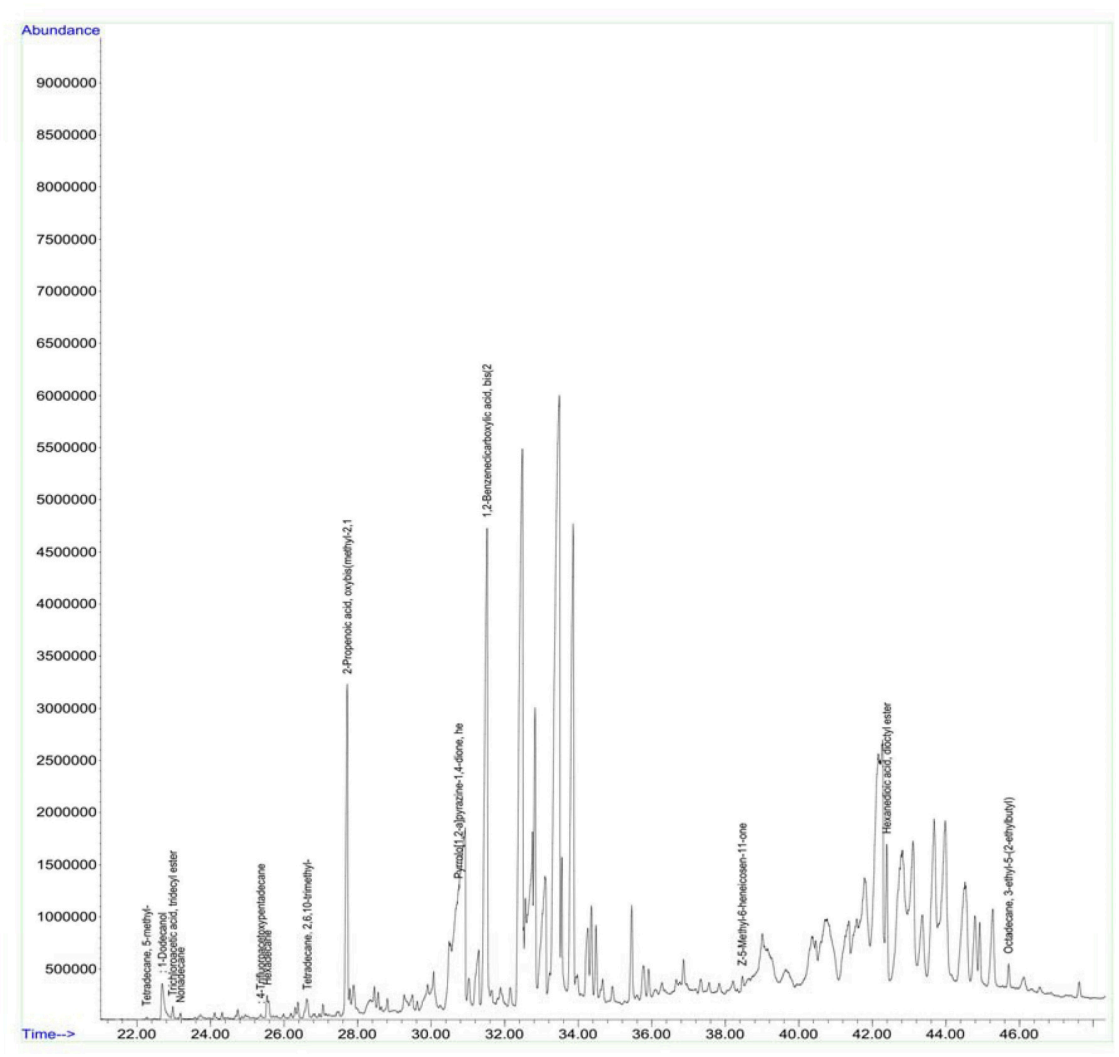
GC chromatogram of the secondary metabolites of *Streptomyces* sp. (A8).

Antimicrobial metabolites including volatile compounds were detected from actinobacteria through standard chromatographic methods (Bucar et al., 2013). Volatile compounds have been reported to exhibit diverse functions such as antibacterial, antifungal, plant growth accelerator or suppressor, infochemical molecules in inter and intra specific interactions, cell-to-cell signaling etc. (Scholler et al., 2002; Cordovez et al., 2015; Schmidt et al., 2015; Zothanpuia et al., 2017). Therefore, the culture filtrates of actinobacterial strains obtained after fermentation were extracted with ethyl acetate and analyzed for volatile compounds by using GCMS. A wide variety of chemical compounds were detected using GCMS library data (Supplementary Table, Figures 8, 9). All the strains showed an array of chemical compounds like amines, acids, pyrrolizidines, ketones, quinones, alcohols, and hydrocarbons. In the present study, production of 1, 2-benzene dicarboxylic acid by all the strains except strain A6 was recorded which may contribute for their antimicrobial potentiality. It has been reported to exhibit anti-extended spectrum β-lactamase activity (Subashini and Krishnan, 2014) as well as anticancer agent against HepG2 and VERO cell lines (Sudha and Selvam, 2012), HepG2 and MCF-7 cell lines (Krishnan et al., 2014). Phenyl ethyl alcohol of strain A2 and 3-phenyl propionic acid from strain A4 are well-known antimicrobial agents against bacteria and fungi (Narayana et al., 2008).

Antimicrobial potential of the strains A1, A3, A5, and A6 may also be due to the elaboration of 2-piperidinone and pyrrolo[1,2-a]pyrazine-1,4-dione from the strains A7 and A8. 2-piperidinone and pyrrolo[1,2-a]pyrazine-1,4-dione are characterized as one of the potential antimicrobials from *Streptomyces* sp. (Khattab et al., 2016) and fungus, *Schistidium antarctici* (Melo et al., 2014), respectively. Heterocyclic compounds like pyrazines are having two nitrogen atoms in their aromatic ring and are reported to have various bioactivities such as antimicrobial, antioxidant, anticancer, neuroprotection against ischemia/reperfusion injuries, and hypoxia (Premkumar and Govindarajan, 2005; Jia et al., 2009; Baldwin et al., 2013; Tan et al., 2015; Ser et al., 2016). Ours is the first report on the production of antimicrobial compounds *viz*., 2-piperidinone by *Dietzia* sp. (A3) and *Kocuria* sp. (A5) and 1, 2-benzene dicarboxylic acid by the former ones including *Nocardiopsis* sp. (A7). Further purification methods need to be standardized to characterize other interesting bioactive compounds produced by these strains.

### Preliminary detection of NRPS and PKS-I biosynthetic gene systems

**Figure 10:**
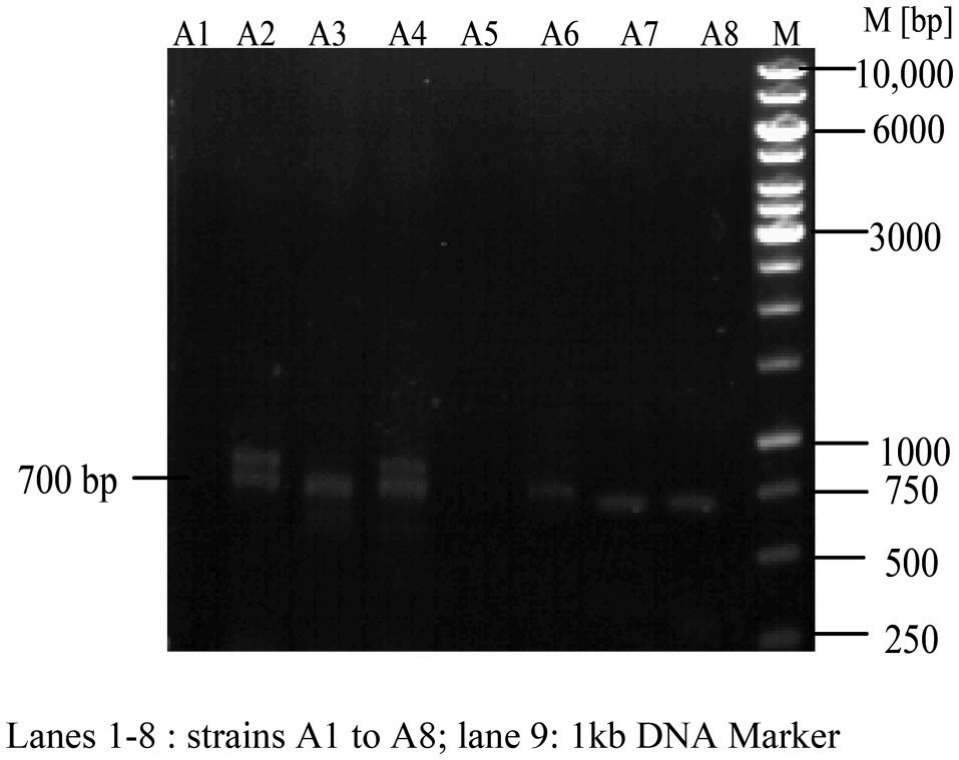
Detection of Non-ribosomal peptide synthetases (NRPS) in actinobacterial strains. NRPS found positive in A2, A3, A4, A6, A7, A8 and negative in the remaining two strains (A1, A5).

Apart from the isolation of antimicrobial compounds through classical extraction methods, genome-based natural product discovery also directs to most possible promising routes for searching novel secondary metabolites from various marine actinobacteria. The synthesis of antimicrobial compounds including polyketide and peptide compounds involves the biosynthetic gene clusters of NRPS and PKS-I, or even the combination of both (Undabarrena et al., 2017). Therefore, an attempt was made to check the presence of these biosynthetic systems in the gDNA of eight strains through PCR specific primers. Out of eight strains, 700 base pair nucleotide fragment specific to NRPS was recorded in the gDNA of six strains (Figure 10) whereas the PKS-I system (1,200–1,400 bp product) was not observed in all the strains tested.

Structurally, biosynthetic systems like NRPS and PKS consist of multifunctional polypeptides with diverse number of modules having different enzymatic properties. The modules of NRPS system constitute the activities related to condensation, adenylation, and thiolation which are mainly concerned in the recognition and condensation of the substrate. On the essentiality of substrate activation, elongation and modification, other domains such as heterocyclase, N-methylase, epimerase, thioesterase, and reductase are also noticed (Ayuso-Sacido and Genilloud, 2005). Degenerate primers A3F and A7R are employed to detect the conserved motif region (700 bp) of adenylation domain. Out of eight strains, six of them showed positive results for NRPS *viz*., A2, A3, A4, A6, A7, and A8 emphasizing the possible mode of production of antimicrobial metabolites through this biosynthetic system.

Similarly, the module of other biosynthetic system, PKS-1 include three domains concerned to ketosynthase (KS), acyltransferase (AT), and acyl carrier protein (ACP) which helps in the selection and condensation of the correct extender unit. They can further act as enoylreductase, dehydratase, and ketoreductase to undergo reduction step of β-keto group formed in the condensation. Systematic coordination of all these domains results in the production of a new polypeptide chain (Donadio and Katz, 1992; Anderson et al., 2000). In the present study, PCR amplification of gDNA of the strains by degenerate PCR primers specific to conserved motif region of PKS-I ketosynthase and methyl-malonyl-CoA transferase (1,200–1,400 bp) was studied. But the expected gene product size was not found in the all the strains tested indicating the probable absence of this system. This may be due to insufficient complementarity of degenerate primers designed for PKS-I module or the bioactivity of the strains may correspond to the metabolites produced through other biosynthetic systems. In secondary metabolism of *Streptomyces* sp. H-KF8, Undabarrena et al. (2017) recorded 26 biosynthetic gene clusters through bioinformatics analysis tool, AntiSMASH and further grouped them into different types *viz*., NRPS, PKS, hybrids, terpenes, RiPP, ectoine, melanine, siderophores, lantipeptides, and butyrolactones.

NRPS synthetases along with fatty acid and/or polyketide synthetases (FAS/PKS) produce different kinds of bioactive compounds including anti-infective, antimicrobial, anticancer etc. For example, pyrrole containing natural products such as prodigiosin, chlorizidine A (anti-tumor), vancomycin (antibacterial), chlorothricin (cholesterol lowering drug) are synthesized through NRPS pathway (Jaremko et al., 2015). In mixed NRPS/PKS pathway, phenyl propionic acids are preferentially activated in the production of microcystins by cyanobacteria (Dickschat, 2011). Kehr et al. (2011) summarized the secondary metabolite pathways in cyanobacteria and stated the key role of NRPS modules in the biosynthesis of unusual signature moiety, 2-piperidone of depsipeptides, Anabaenopeptilides. In the present study, various volatile compounds including pyrrole, and piperidinone derivatives, phenyl propionic acid, carboxylic acids etc. were detected in the biologically active crude extracts of eight potent actinobacterial strains isolated from East Coast of Andhra Pradesh, India through GCMS which may contribute for their antimicrobial potential using NRPS biosynthetic pathway. Further investigations should be performed to purify the antimicrobial metabolites of the strains through analytical (chromatography, spectroscopy) techniques and also to elucidate their actual biosynthetic pathways.

## Conclusion

Out of 73 marine actinobacterial strains obtained from different coastal regions of India, eight of them isolated from East Coast of Andhra Pradesh exhibited strong antimicrobial potential. Six actinobacterial strains (A1, A2, A3, A4, A6, and A7) showed high yields of antimicrobial metabolites on 5th day of incubation whereas the other two strains (A5 and A8) exhibited after 6 days. ISP-2 (for strains A1, A3, A4, A5, A6), ISP-1 (for strains A7 and A8) and Starch casein (for strain A2) supported the production of antimicrobials. Preferential utilization of carbon sources by the strains was widely varied. Maximum yields of antimicrobial metabolites were observed in optimal culture media amended with different carbon sources like xylose (for strains A1, A3, and A7), starch (for strains A2 and A5), dextrose (for strain A4), mannitol (for strain A6), and galactose (for strain A8). Nitrogen sources such as malt extract and yeast extract served as the best candidates for antimicrobial potential of most of the strains while the strains A7 and A8 proved their efficacy in the optimal culture media with tryptone amendment. Detection of volatile compounds *viz*., 1, 2-benzene dicarboxylic acid from most of the actinobacterial strains, 2-piperidinone, and pyrrolo[1,2-a]pyrazine-1, 4-dione from few of them through GCMS suggests that they may contribute for their major antimicrobial potential. Analysis of biosynthetic gene systems for the production of antimicrobials revealed the presence of NRPS in the strains A2, A3, A4, A6, A7, and A8. Further study need to be done to predict and isolate potent bioactive compounds from the actinobacterial strains of East Coast of Andhra Pradesh, India.

## Author contributions

AK designed and performed whole experimental part of the work in HSS lab, Indian Institute of Science, Bangalore, India.

### Conflict of interest statement

The authors declare that the research was conducted in the absence of any commercial or financial relationships that could be construed as a potential conflict of interest.
