# Corrigendum: Biological Significance of Marine Actinobacteria of East Coast of Andhra Pradesh, India

**DOI:** 10.3389/fmicb.2020.00074

**Published:** 2020-02-04

**Authors:** Alapati Kavitha, Handanahal S. Savithri

**Affiliations:** Department of Biochemistry, Indian Institute of Science, Bangalore, India

**Keywords:** marine actinobacteria, *Dietzia*, *Kocuria*, *Nocardiopsis*, *Streptomyces*

In the original article, there was an error in printing the order of Polymerase Chain Reaction (PCR) conditions. “Thermal cycling was carried out with a model S1000 (Bio-Rad, USA) and all the samples were subjected to an initial denaturation at 98°C for 3 min, followed by 28 consecutive cycles of annealing (1 min at 52°C), extension (2 min at 72°C) and denaturation (1 min at 94°C). A final extension step at 72°C for 5 min was also included at the end”. A correction has been made to “Materials and Methods”, “Molecular Identification of the Potent Actinobacterial Strains Through Genomic (16S rRNA Gene Fragment) Analysis”, “Page Number 3, Second paragraph”:

“Thermal cycling was carried out with a model S1000 (Bio-Rad, USA) and all the samples were subjected to an initial denaturation (3 min at 98°C) followed by denaturation (1 min at 94°C), annealing (1 min at 52°C, 28 consecutive cycles), extension (2 min at 72°C), and a final extension (5 min at 72°C) step at the end.”

The authors apologize for this error and state that this does not change the scientific conclusions of the article in any way. The original article has been updated.

