# Supplementary material for: Biological Significance of Marine Actinobacteria of East Coast of Andhra Pradesh, India
Source: Front Microbiol. 2017 Jul 6;8:1201. doi: 10.3389/fmicb.2017.01201 (PMC5498559; doi:10.3389/fmicb.2017.01201)
Supplement: Supplementary file 1 [file DataSheet1.pdf]

Supplementary Table. Volatile compound analysis by Gas Chromatography Mass Spectra (GCMS).

S.No.	Actinobacterial strains	GCMS products	Retention time
1.	A1	1,8-Nonadien-3-ol	10.827
		1-Hexanol, 2-ethyl-	11.923
		6-Hydroxymethyl-5-methyl-bicycl	13.529
		1,8-Nonadien-3-ol	14.231
		Tin, dichlorodimethyl-	14.929
		Acetic acid, 2-ethylhexyl ester	15.708
		DL-Serine, N-[(phenylmethoxy)ca	16.372
		1H-Indene, 1-methylene-	16.629
		2-Piperidinone	18.462
		DL-Leucine, N-glycyl-	19.031
		Caprolactam	19.416
		2-Trifluoroacetoxydodecane	19.571
		Bicyclo[4.4.1]undeca-1,3,5,7,9-	19.879
		Tetradecane	20.007
		Benzoic acid, 2-methylpropyl es	20.881
		2(3H)-Naphthalenone, 4,4a,5,6,7	21.288
		Chloroacetic acid, 2-ethylhexyl	21.694
		9-Methyltricyclo[4.2.1.1(2,5)	21.994
		Biphenyl	22.204
		Benzenepropanoic acid, dodecyl	22.525
		2-Ethylhexyl mercaptoacetate	23.621
		Tetradecane, 2,6,10-trimethyl-	24.285
		tert-Hexadecanethiol	24.803
		8-Heptadecene, 9-octyl-	25.009
		1,2-Benzenedicarboxylic acid, d	47.617
2.	A2	1,8-Nonadien-3-ol	12.659
		Phenylethyl Alcohol	13.177
		Tin, dichlorodimethyl-	13.588
		Acetic acid, 2-ethylhexyl ester	14.034
		Glutaconic acid	14.261
		Bicyclo[4.4.1]undeca-1,3,5,7,9-	18.050
		d-Mannose	18.380
		1,4-Dioxaspiro[4.5]decane-7-m	18.504
		E-2-Hexenyl benzoate	18.997
		Trichloroacetic acid, 2-ethylhe	19.780
		Octadecane, 6-methyl-	20.752
		2-Ethylhexyl mercaptoacetate	21.669
		Tetradecane, 2,6,10-trimethyl-	22.324
		tert-Hexadecanethiol	22.846
		8-Heptadecene, 9-octyl-	23.039
		Butylated Hydroxytoluene	23.677
		2,5-Octadecadiynoic acid, met	24.268
		Falcarinol	25.415
		Methyl 5,7-hexadecadiynoate	26.464
		Propanoic acid, 2-methyl-, (dec	27.976
		1H-2,8a-Methanocyclopenta[a]cyc	28.567
		1,2-Benzenedicarboxylic acid,	45.609
3.	A3	1,8-Nonadien-3-ol	12.638
		Acetic acid, 2-ethylhexyl ester	14.030
		Tin, dichlorodimethyl-	14.154

		2-Piperidinone, 1-methyl-	14.261
		11-Oxa-dispiro[4.0.4.1]undecan-	17.729
		Benzeneacetic acid, 4-tetradecy	18.127
		Propanedioic acid, phenyl-	18.273
		Nonanoic acid	18.273
		1-Propanol, 3-(phenylmethoxy)-	19.001
		Benzenepropanoic acid, pentadec	19.793
		Benzenepropanoic acid	20.435
		Benzenepropanoic acid, tridecyl	20.748
		2-Ethylhexyl mercaptoacetate	21.669
		Tetradecane, 2,6,10-trimethyl-	22.320
		1-Dodecanol, 3,7,11-trimethyl-	22.846
		8-Heptadecene, 9-octyl-	23.035
		tert-Hexadecanethiol	23.236
		4,6-di-tert-Butyl-m-cresol	23.677
		11,13-Dihydroxy-tetradec-5-ynoi	25.415
		Methyl 5,7-hexadecadiynoate	26.379
		10,13-Octadecadiynoic acid, met	26.683
		Doconexent	27.141
		2-Propenoic acid, oxybis(methyl	27.813
		Cinnamic acid, 4-hydroxy-3-meth	30.190
		1,2-Benzenedicarboxylic acid,	31.911
		1,2-Benzenedicarboxylic acid, b	32.832
		1,2-Benzenedicarboxylic acid, d	45.571
4.	A4	1-Hexanol, 2-ethyl-	10.642
		6-Hydroxymethyl-5-methyl-bicycl	12.064
		Nonanal	12.689
		Tin, dichlorodimethyl	13.340
		Acetic acid, 2-ethylhexyl ester	14.034
		4-[Dichloromethyl]-2-[[2-[1-m	14.034
		3-Trifluoroacetoxydodecane	17.691
		Bicyclo[4.4.1]undeca-1,3,5,7,9-	18.037
		Benzeneacetic acid, 2-tetradecy	18.123
		Benzoic acid, 2-methylpropyl es	18.992
		2(3H)-Naphthalenone, 4,4a,5,6,7	19.570
		Chloroacetic acid, 2-ethylhexyl	19.780
		3-Phenyl-propionic acid, isopro	20.285
		Tetradecane, 2,6,10-trimethyl-	20.744
		2-Ethylhexyl mercaptoacetate	21.681
		tert-Hexadecanethiol	22.850
		8-Heptadecene, 9-octyl-	23.043
		4,6-di-tert-Butyl-m-cresol	23.681
		2,5-Octadecadiynoic acid, methyl	24.135
		Methyl 5,7-hexadecadiynoate	24.281
		Falcarinol	25.432
		1,2-Benzenedicarboxylic acid,	33.221
		1,2-Benzenedicarboxylic acid,	45.746
5.	A5	1,8-Nonadien-3-ol	10.647
		w-Isonitrosoacetophenone	11.623
		4-[Dichloromethyl]-2-[[2-[1-met	12.068
		Nonanal	12.698
		Tin, dichlorodimethyl-	13.147
		Acetic acid, 2-ethylhexyl ester	14.034
		1H-Indene, 1-methylene-	14.950
		2-Piperidinone	17.151
		Bicyclo[4.4.1]undeca-1,3,5,7,9-	18.046

		Octadecane, 6-methyl-	18.127
		Benzeneacetic acid	18.765
		1-Propanol, 3-(phenylmethoxy)-	18.765
		2(3H)-Naphthalenone, 4,4a,5,6,7	19.562
		Benzeneacetic acid, 4-tetradecy	19.780
		2-Ethylhexyl mercaptoacetate	21.699
		Tetradecane, 2,6,10-trimethyl-	22.328
		tert-Hexadecanethiol	22.850
		8-Heptadecene, 9-octyl-	23.047
		4,6-di-tert-Butyl-m-cresol	23.681
		[5,9-Dimethyl-1-(3-phenyl-oxira	24.126
		5-Benzofuranacetic acid, 6-ethe	25.278
		2-[4-methyl-6-(2,6,6-trimethylc	25.432
		1,2-Benzenedicarboxylic acid, b	33.256
		1,2-Benzenedicarboxylic acid,	45.703
6.	A6	1-Hexanol, 2-ethyl-	10.638
		Nonanal	12.694
		Acetic acid, 2-ethylhexyl ester	14.034
		Tertbutyloxyformamide, N-methyl	14.291
		N-Methyl-9-aza-tricyclo[6.2.2.0	14.950
		2-Piperidinone	16.205
		Benzoic acid, 2-methylpropyl es	19.001
		Chloroacetic acid, 2-ethylhexyl	19.789
		Benzenepropanoic acid, pentadec	20.915
		2-Ethylhexyl mercaptoacetate	21.707
		Geranyl isovalerate	22.332
		tert-Hexadecanethiol	23.047
		4,6-di-tert-Butyl-m-cresol	23.690
		[5,9-Dimethyl-1-(3-phenyl-oxi	24.144
		Methyl 5,7-hexadecadiynoate	24.884
		Hexa-t-butylselenatrisiletane	45.905
**7.**	A7	6-Ethyl-3-(1-methylethyl)tetrah	21.018
		1,8-Nonadien-3-ol	22.251
		1-Dodecanol	22.675
		Trichloroacetic acid, tridecyl	22.983
		Nonadecane	23.184
		3-Trifluoroacetoxypentadecane	23.570
		Phenol, 2,4-bis(1,1-dimethyleth	23.733
		Hexanoic acid, 4-tridecyl ester	24.178
		trans-2-undecenoic acid	24.709
		Hexadecane	25.535
		Tetradecane, 2,6,10-trimethyl-	26.828
		p-Octylacetophenone	26.987
		1-Hexadecanol, 2-methyl-	27.770
		2-Propenoic acid, oxybis(methyl	27.775
		Octadecane, 1-chloro-	27.899
		Pyrrolo[1,2-a]pyrazine-1,4-dion	31.462, 42.286
		1,2-Benzenedicarboxylic acid	31.564
		a-D-Xylofuranose, cyclic 1,2:3,	34.155
		2,5-di-tert-Butyl-1,4-benzoquin	34.626
		9-Octadecenethioic acid, 12-hyd	34.789
		17-Pentatriacontene	38.231
		1,9-Dioxacyclohexadeca-4,13-d	39.529
		Ergotaman-3',6',18-trione, 9,10	41.528
		Octadecane, 3-ethyl-5-(2-ethylbutyl)	45.721

8.	A8	Tetradecane, 5-methyl-	22.251
		1-Dodecanol	22.688
		Trichloroacetic acid, tridecyl	22.974
		Nonadecane	23.180
		4-Trifluoroacetoxypentadecane	25.372
		Hexadecane	25.535
		Tetradecane, 2,6,10-trimethyl-	26.640
		2-Propenoic acid, oxybis(methyl	27.719
		Pyrrolo[1,2-a]pyrazine-1,4-dion	30.742, 31.294
		1,2-Benzenedicarboxylic acid, b	31.521
		Z-5-Methyl-6-heneicosen-11-one	38.467
		Hexanedioic acid, dioctyl ester	42.393
		Octadecane, 3-ethyl-5-(2-ethylb	45.703
